# Activity-dependent decrease in contact areas between subsurface cisterns and plasma membrane of hippocampal neurons

**DOI:** 10.1186/s13041-018-0366-7

**Published:** 2018-04-16

**Authors:** Jung-Hwa Tao-Cheng

**Affiliations:** NINDS Electron Microscopy Facility, National Institute of Neurological Disorders and Stroke, National Institutes of Health, Bethesda, MD 20892 USA

**Keywords:** ER-PM contact sites, Calcium regulation, Electron microscopy

## Abstract

Subsurface cistern (SSC) in neuronal soma and primary dendrites is a specialized compartment of endoplasmic reticulum (ER) that is in close apposition (10 nm) with the plasma membrane (PM). ER-PM contact areas are thought to be involved in intracellular calcium regulation. Here, structural changes of SSC in hippocampal neurons were examined by electron microscopy upon depolarization with high K^+^ (90 mM) or application of NMDA (50 μM) in rat dissociated cultures as well as organotypic slice cultures. The number and average length of SSC-PM contact areas in neuronal somas significantly decreased within 30 s under excitatory condition. This decrease in SSC-PM contact area progressed with time and was reversible. These results demonstrate a structural decoupling between the SSC and the PM upon stimulation, suggesting that there may be a functional decoupling of the calcium regulation. Because SSC-PM contact areas may mediate calcium influx, the decrease in contact area may protect neurons from calcium overload upon heightened stimulation.

**Electronic supplementary material:**

The online version of this article (10.1186/s13041-018-0366-7) contains supplementary material, which is available to authorized users.

## Introduction

Specialized contact areas between endoplasmic reticulum (ER) and plasma membrane (PM) are termed as ER-PM connections, contact sites, or junctions, which have well-defined structural characteristics and are involved in calcium regulation and lipid transport [1, 2]. One of the most studied functions of ER-PM contact areas is store-operated calcium entry (SOCE), a fundamental signaling mechanism in majority of cell types including neurons [3, 4]. SOCE is initiated by lowered Ca^2+^ levels in ER (store depletion) that leads to Ca^2+^ influx from extracellular milieu at PM-ER junctions, and eventually to restore Ca^2+^ levels in ER.

Store depletion also induces increased formation of ER-PM contact areas, and this dynamic process is often visualized by live observation of overexpressed STIM1 (stromal interaction molecule 1), an ER protein closely involved in SOCE [5]. ER-PM contact areas were also induced by overexpression of extended synaptotagmins [6] and TMEM24 [7], two groups of ER protein unrelated to SOCE, as well as by overexpression of a PM protein, KV2.1, a voltage-gated potassium channel [8]. Thus, ER-PM contact can be induced by different mechanisms as well as by overexpression of different proteins in many cell types. However, few such studies [3, 8] were carried out in neurons.

The ER-PM contact areas in neurons display unique ultrastructural features by electron microscopy (EM) [9], and the term “subsurface cistern” (SSC) is specific to such ER in neurons [10]. In addition to SOCE-related proteins [3], neurons contain additional channels and receptors involved in calcium regulation at the SSC-PM contact areas such as voltage-dependent calcium channels Cav2.1, Cav1.2, and calcium-activated potassium channels, BK and SK2, as well as IP3 and ryanodine receptors [8, 11–13]. Thus, SSC-PM structural coupling may represent a more complicated form of calcium regulation specific to neurons. Interestingly, neuronal SSC is also associated with concentrated labeling of Kv2.1 [14], and that the KV2.1 clusters are disrupted and decrease in number by neuronal activity [15]. Furthermore, observation of live neurons with overexpressed Kv2.1 as well as ER proteins showed dissociation of SSC from the PM upon application of glutamate [8]. However, detailed ultrastructural data on the dynamics of SSC-PM dissociation in neurons is still lacking.

The present EM study set out to demonstrate activity-dependent structural changes in SSC-PM contact areas in neurons with endogenous proteins without overexpression of proteins. Two experimental systems, dissociated and organotypic slice cultures of the hippocampus, where excitatory stimulations can be easily applied and manipulated were used. SSCs in these neurons were classified according to structural characteristics, and the number and length of all SSCs were measured under basal and excitatory conditions to document the time course and extent of the activity-induced changes in the SSC-PM contact areas.

## Methods

### Preparation, treatment and fixation of rat dissociated hippocampal neuronal

Cell cultures were prepared as described before [16] from embryonic 20-day-old rat fetuses by papain dissociation, and then plated onto previously prepared rat glial feeder cultures. The cultures were kept in 10% CO_2_ incubator at 35 °C, and experiments were carried out with three-week-old cultures.

Culture dishes were placed on a floating platform in a water bath maintained at 37 °C for all experiments. Control incubation medium was HEPES-based Krebs Ringer at pH 7.4. High K^+^ medium was at 90 mM KCl, with osmolarity compensated by reducing the concentration of NaCl. N-methyl-D-aspartic acid (NMDA) medium contained 30–50 μM NMDA in the control medium. Cell cultures were washed with control medium and treated for 2 min with either control or high K^+^ media, or with 2–3 min with NMDA, and then fixed immediately. To examine recovery after depolarization with high K^+^, samples were washed 4–5 times with control medium within two min after removing the high K^+^ medium, then fixed with 4% glutaraldehyde in 0.1 N cacodylate buffer at pH 7.4 for 30 min at room temperature and then stored at 4 °C.

### Preparation, treatment and fixation of rat organotypic hippocampal slice cultures

All samples were from a previously published report [17] and reexamined here tor structural changes of the SSC. Briefly, the hippocampus was removed from postnatal 6–8 day old rats and cut at 250 μm thickness with a tissue chopper. Slices were placed on a cell culture inserts in six-well culture dishes and incubated in culture media in a 5% carbon dioxide incubator at 35 °C. Slices were used 10–14 days in vitro.

Slice culture inserts in six-well dishes were placed on a floating platform in a water bath at 37 °C. Normal, high K^+^ and NMDA medium were the same as used for dissociated cells. Both high K^+^ and NMDA treatments were for 0.5, 1, 2, 3, or 5 min. To examine recovery after depolarization, high K^+^ medium was removed and the samples were washed three to four times in normal incubation medium for a total of 1, 2, 5, 10, 30 and 60 min. Experimental controls were processed in parallel, including all the medium changes and washing steps. Slice cultures were fixed with 2% glutaraldehyde and 2% paraformaldehyde, or 4% glutaraldehyde in 0.1 N cacodylate buffer at pH 7.4 for 1–3 h at room temperature and then stored at 4 °C.

### Electron microscopy

Fixed samples were washed in buffer, treated with 1% osmium tetroxide in 0.1 N cacodylate buffer at pH 7.4 for 1 h on ice, washed and en bloc stained with 0.25–1% uranyl acetate in 0.1 N acetate buffer at pH 5.0 overnight at 4 °C, dehydrated with a series of graded ethanol, and finally embedded in epoxy resins. Thin sections (70–90 nm) were cut, counter stained with uranyl acetate and lead citrate, and examined on a JEOL1200 EX transmission electron microscope at 60 KV accelerating voltage. Images were collected with a digital CCD camera (AMT XR-100, Danvers, MA, USA) at 15,000×.

### Morphometry

For dissociated hippocampal cultures, every neuronal soma encountered was examined along the plasma membrane for evidence of subsurface cistern (SSC). For organotypic hippocampal slice cultures, sampling was restricted to the pyramidal neurons of the CA1 area. Although SSC were present in neuronal soma and primary dendrites [9, 10], the present study restricted sampling for morphometry to somas only because many dendrites can arise from a single neuron, and sampling from dendrites may include multiple data points collected from the same neuron. In contrast, somas are easy to identify individually, and thus, the simplest way to ensure that each data point was from a separate neuron.

**Fig. 1:**
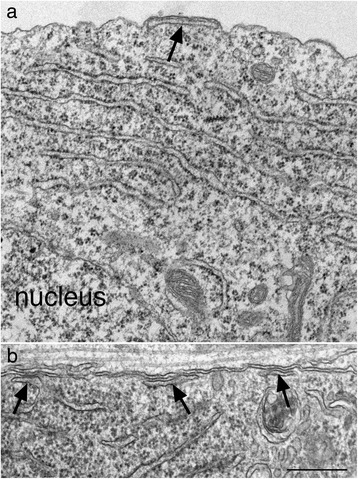
Electron micrographs of subsurface cisterns (SSC, marked by arrows) sampled from dissociated cells (**a**) and slice cultures (**b**). Scale bar = 0.5 μm

**Fig. 2:**
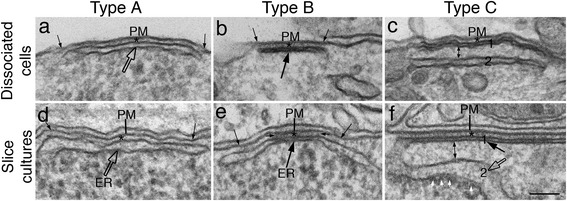
Three types of SSC are classified in dissociated hippocampal neurons (top panels) and in hippocampal slice cultures (lower panels): Type A – a single stack of ER with an open cistern (open arrows in **a** & **d**). Type B – a single stack of ER with a segment of flattened cistern (large black arrows in **b** & **e**). There is barely any discernible lumen in the flattened segment, and the gap between the flattened segment and PM is filled with dark material (area between two short, small arrows in **e**). Type C – double stacks of ER with a flattened first stack (1) beneath the PM, and a second stack (2) below (1). The gap between the two stacks is filled with amorphous materials and the ER membranes facing the gap are typically devoid of ribosomes (white small arrows in **f**) that typically are attached on the ER membrane facing the cytoplasm. The distance between the two ER stacks are variable, ranging from 30 to 60 nm, in both culture systems. For example, the distance (double-headed arrow) is ~ 40 nm in (**c**), and ~ 60 nm in (**f**). In all three types of SSC, the gap between the first stack of ER and the PM is uniform at 10 nm (*), and the length of SSC is measured between the two long, small arrows on the PM in (**a**, **b**, **d**, **e**). Scale bar = 0.1 μm

SSC is defined as an ER cistern closely apposed to the plasma membrane (PM) with a relatively uniform distance of 10 nm between the ER and the PM (arrows in Fig. 1). Every SSC encountered in neuronal somas was photographed and counted. At least 10 somal profiles were scored for each sample, with more than 200 somas scored from 15 samples of dissociated cultures and more then 500 somas scored form 30 samples of slice cultures. Neurons that were partially under the grid bar were still included in the sampling as long as at least half of the nucleus was visible. Due to the fact that many neurons were scored with partial profiles, the total number of SSC from each sample was pooled from all somas that were scored, and then divided by the number of neurons and normalized as number of SSCs per 10 neuronal somas. This practice ensures that each sample is treated the same, without individual number on each neuron but resulting in one data point per sample. The length of SSC is the linear measurement of the PM-SSC contact area where the two membranes are rigidly apposed (e. g., the edges of the contact area are indicated by two small arrows on the PM in Fig. 2a, b, d, e). The average length of SSC is presented as mean ± SEM with SD also indicated for each sample. Comparison between two groups of samples was tested by Student t test or paired t test. Comparisons among three groups of samples were tested by ANOVA.

## Results

### Ultrastructure of subsurface cisterns (SSCs)

SSC in neurons is composed of an ER stack closely apposed to the plasma membrane. Examples of SSCs in hippocampal dissociated cultures (Fig. 1a) and in organotypic slice cultures (Fig. 1b) shared similar structural features. Three types of SSCs were classified based on structural characteristic (Fig. 2) and the total number of SSC measured in the present study includes all three types of SSC.

### The SSC-PM contact area significantly decreased upon excitatory stimulation in dissociated rat hippocampal neuronal cultures

**Fig. 3:**
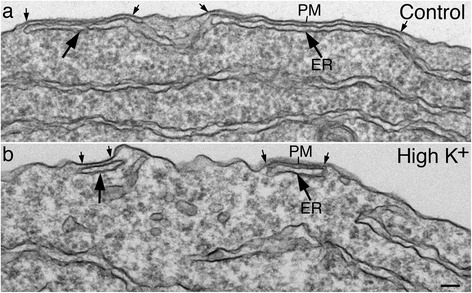
Electron micrographs of dissociated hippocampal neuronal somas under control condition (**a**) and upon depolarization with high K^+^ (**b**). Large arrows point to subsurface cisterns where the endoplasmic reticulum (ER) comes in close contact with the plasma membrane (PM). The contact area between ER and PM (small arrows point to the borders of the contact area) conspicuously decreased upon depolarization. Scale bar = 0.1 μm

**Fig. 4:**
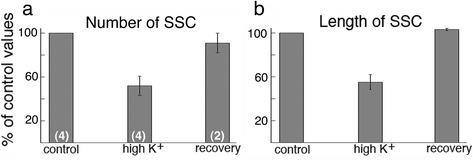
Both the total number of SSC (**a**) and the average length of SSC (**b**) significantly decreased upon depolarization with high K^+^ (90 mM, 2 min) and returned to control values upon 30 min of recovery in control medium. (n) = number of experiments listed on the bottom of bars in (**a**). Complete data from all experiments are listed in Additional files 1 and 2 (exp 1–4). For number of SSC: *P* < 0.005, control vs. K; *P* < 0.05, K vs. recovery by ANOVA. For length of SSC: *P* < 0.0005, control vs. K; *P* < 0.005, K vs. recovery by ANOVA

One striking observation is that the SSC-PM contact area in neuronal somas conspicuously decreased upon depolarization with high K^+^ (Fig. 3). Total number of SSC decreased to 52 ± 8.7%, and returned to 91 ± 9% of control values upon 30 min of recovery in control medium (Fig. 4a). The average length of SSC decreased to 55 ± 6.9%, and returned to 103 ± 1% of control values upon recovery (Fig. 4b). When combining the effects of both the number and length of the SSC, there was less than 30% of control values remaining upon depolarization.

In order to see if NMDA receptor is involved in this structural change of SSC-PM contact, cells were treated with NMDA (30–50 μM, for 2–3 min). Similar to depolarization with high K^+^, NMDA treatment resulted in significant decreases in both the total number of SSC as well as the average length of SSC (exp 5 and 6 in Additional files 1 and 2).

### The SSC-PM contact area significantly decreased upon excitatory stimulation in pyramidal neurons of the CA1 region from hippocampal slice cultures

In contrast to dissociated hippocampal cultures which contain a mixture of neuronal cell types (e. g., pyramidal neurons from CA1, CA2 and CA3 areas, and granule cells from the dentate gyrus, as well as inhibitory interneurons), organotypic slice cultures maintain the anatomical arrangement of the different cell types, retaining the layers that resemble their locations in vivo [18]. Here, the focus is on the pyramidal neurons of the CA1 region to see whether the structural changes observed in dissociated neurons can be verified in slice cultures.

#### Total number of SSC decreased upon stimulation

**Fig. 5:**
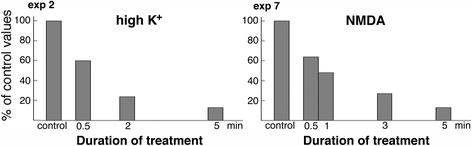
Total number of SSC in pyramidal neurons of the CA1 region of hippocampus from slice cultures decreased progressively upon excitatory stimulation (plotted from two experiments in Additional file 3)

The total number of SSC in slice cultures (100 ± 9 per 10 neurons under control conditions, 8 experiments, Additional file 3) were similar to that in dissociated cells (96 ± 18, 6 experiments, Additional file 1). The total number of SSCs in slice cultures significantly decreased upon depolarization with high K^+^ as well as treatment with NMDA (Additional file 3), and this decrease progressed with time of treatment. Two representative experiments are shown as bar graphs in Fig. 5.

Interestingly, upon similar excitatory stimulations, the decrease in SSC numbers appeared to be more pronounced in slice cultures than in dissociated cells. For example, the average decrease upon depolarization or NMDA treatment was ~ 67% (exp 2, 5, 6 in Additional file 3) and 60% (exp 7–8 in Additional file 3), respectively, in slice cultures, vs. ~ 48% (exp 1–4 in Additional file 1) and 36% (exp 5–6 in Additional file 1) in cells.

#### The decrease in total number of SSC upon depolarization is reversible

**Fig. 6:**
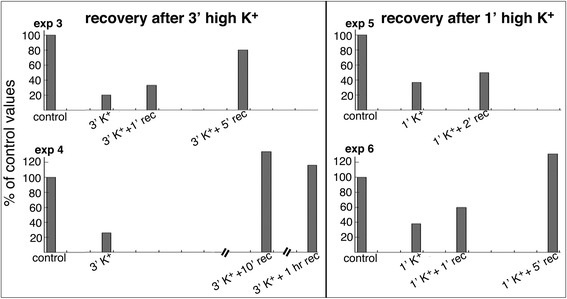
The decrease in total number of SSC in slice cultures upon depolarization with high K^+^ is reversible (plotted from exp. 3–6 in Additional file 3)

When the slice cultures were returned to control medium after depolarization with high K^+^, the total number of SSC gradually returned to control levels, possibly with an overcompensation at times (e. g., Additional file 3, exp. 4 and 6). As expected, the time required for recovery was longer after a longer high K^+^ treatment. It took 10 min to recover after 3 min of high K^+^, while 5 min of recovery is sufficient after 1 min of high K^+^ (Fig. 6).

#### The average length of SSC decreased upon depolarization

**Fig. 7:**
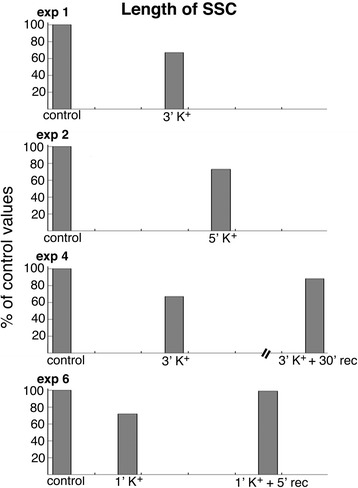
The average length of SSC decreased to 67–73% of control values upon 1–5 min of depolarization with high K^+^, and returned to 88–99% of control values upon recovery (data listed in Additional file 4)

To verify whether the contact area between SSC and PM also reduced in size in slice cultures, the length of SSC from four representative experiments was listed in Additional file 4 and plotted in Fig. 7. The average length of SSC consistently decreased upon depolarization with high K^+^, and returned to near control levels after recovery in control medium.

The average length of SSC under control conditions was shorter in slice cultures (263 ± 5 nm) than in dissociated cells (355 ± 31 nm), and the maximal range of length of SSC is also much shorter in slice cultures (1.3 μm, Additional file 4) than in dissociated cells (2.5 μm, Additional file 2). Upon similar protocol of depolarization with high K^+^, the percent decrease in length of SSC is less in slice cultures (~ 30%, Additional file 4) than in dissociated cells (~ 45%, Additional file 2). For slice cultures, when multiplying the percent values of both the number (~ 33%, Additional file 3) and length (~ 70%, Additional file 4) of the SSC, there were less than 25% of control values remaining upon depolarization.

### The SSC with a flattened cistern (type B & C) is more stable than SSC with an open cistern (type A)

In order to assess whether the three types of SSC classified in Fig. 2 were differentially affected by depolarization, the percentages of each type were calculated for each experiment. The combined results from 4 experiments each from dissociated cells and slice cultures are listed in Additional file 5 as mean ± SEM. In both culture systems, the great majority of SSCs are Type A, the type with one open cistern (Fig. 2a, d) whether under control or upon depolarization with high K^+^.

Although the trend that the total number of SSC decreased upon stimulation was the same in both dissociated and slice cultures, the proportion of different types of SSC in the two systems was different. Under control conditions, there were more SSCs of types B & C with a flattened cistern of ER in the slice cultures (15.2%, Type B + Type C in Additional file 5) than in the dissociated culture (4.3%), indicating that the growth conditions of the slice culture may be more conducive for the formation of SSC with a flattened cistern. This higher presence of the SSC with a flattened cistern made it easier to measure any difference in their distribution upon depolarization in slice cultures than in dissociated cells. When the percentage change was calculated separately for SSC with a flattened cistern (Additional file 6), it is clear that upon stimulation, these SSCs also decreased in number, but to a lesser extent than the total number of SSC. Thus, the SSCs with a flattened cistern may be more stable than those with an open cistern.

## Discussion

The present EM study provided measurements at the ultrastructural level that SSC-PM contact areas significantly decreased in hippocampal neurons upon excitatory stimulation. This finding is in agreement with a previous light microscopy study where fluorescence-tagged ER dissociated from PM in dissociated hippocampal neurons upon application of glutamate [8]. In the present study, the structural decoupling between SSC and PM occurred within 30 s after depolarization with high K^+^ or application of NMDA, progressed with treatment time, and was reversible upon return to control conditions.

One of the striking features of ER-PM junction in all cell types is the relatively uniform distance between the two apposing membranes within each junction. The thickness and morphological characteristics of the junctional materials are dependent on specific tethering molecules, such as STIM1 and extended synaptotagmin present in the ER, and Orai1 in the PM [5, 19, 20]. In neurons, additional proteins at these junctions likely contribute to the especially dark material in the gap between the PM and the flattened segment of the ER (SSCs of types B & C), probably making these junctions structurally more stable. Interestingly, prevalence of SSC with a flattened segment varied widely in different types of neurons. For example, it is virtually the exclusive type in cerebellar Purkinje neurons where some calcium and potassium channels are located in clusters on the apposed PM [11, 12]. However, in hippocampal pyramidal neurons studied here, it only represents 5–15% of the SSCs, an occurrence frequency too low to correspond to all the labeled clusters of calcium and potassium channels [8, 21]. Thus, it is likely that the PM atop of SSCs with an open cistern also contain concentrated calcium and potassium channels in pyramidal neurons.

In addition to the common type of ER-PM junction with one single stack of ER, multiple stacks of ER of uniform spacing can form in HeLa cells with overexpression of STIM1 [19]. Interestingly, neurons can form multiple stacks of ER at SSC-PM junctions without overexpression of proteins, and junctions with three or more stacks of ER are fairly common in adult brains [9, 10, 22]. In these SSC-PM junctions, the distance between the PM and the first stack of ER is at 10 nm, the same as reported here in the two tissue culture systems. However, the distance between the subsequent multiple stacks of ER in adult brains is constant at 30 nm, in contrast to the gap that ranged from 30 to 60 nm in the two culture systems studied here.

In addition to structural complexity, the composition of the neuronal type of SSC-PM junction is also different from that of other cell types. Since the first suggestion of neuronal SSC’s role in calcium-related activity near plasma membrane [23], many proteins involved in calcium regulation have been localized at these SSC-PM junctions by immunolabeling [8, 11–13]. These findings indicate a specialized domain at these SSC-PM junctions unique to neurons, and calcium regulation at these sites is more complicated than SOCE that is ubiquitous in most cells. In the least, it is possible that in neurons, calcium influx may occur more efficiently at these specialized locations due to the high concentration of voltage-dependent calcium channels.

In neurons, excitatory stimulation leads to calcium influx via voltage-dependent calcium channels as well as NMDA receptors, and the resulting calcium influx dominates over SOCE in calcium regulation for this cell type [4]. The present study demonstrated that activation of NMDA receptors is involved in the structural changes of SSC-PM contacts. However, the mechanism is still unclear. The possibilities include action potential induced by NMDA treatment, direct calcium influx via NMDA receptor in the soma, or calcium influx via voltage dependent calcium channel induced by activation of postsynaptic NMDA receptors.

The intracellular calcium rise in neurons is dependent on the intensity and duration of the stimuli, and extensive calcium rise is detrimental to neurons [24]. In line with the suggestion that a disruption of the calcium-sensitive, SSC-associated Kv2.1 clustering may offer neuroprotection [25], the significant decrease in SSC-PM contact area reported here may represent a mechanism to protect neurons from calcium overload by reducing concentrated calcium influx at these sites during heightened stimulation.

## Additional files

Additional file 1: Number of subsurface cisterns (SSC) normalized to per 10 neuronal somas in dissociated hippocampal cultures. (PDF 264 kb)

Additional file 2: Average length of subsurface cistern (mean ± SEM in nm) in neuronal somas of dissociated hippocampal neuronal cultures. (PDF 46 kb)

Additional file 3: Number of subsurface cistern (SSC) normalized to per 10 pyramidal neuronal somas in the CA1 region of the hippocampus in organotypic slice cultures. (PDF 264 kb)

Additional file 4: Average length of subsurface cistern (mean ± SEM in nm) in pyramidal neuronal somas in the CA1 region of organotypic hippocampal slice cultures. (PDF 45 kb)

Additional file 5: Percentages of each of the three types of SSC (A, B and C) in neuronal somas before and after depolarization. (PDF 264 kb)

Additional file 6: (A) Total number of SSC and (B) Subtotal number of SSC with a flattened stack of ER in pyramidal neuronal somas in the CA1 region of hippocampal slice cultures. Number of SSC in control samples normalized to per 10 neuronal somas. Values for other conditions normalized to % of controls. (PDF 52 kb)
