# Supplementary material for: Activity-dependent decrease in contact areas between subsurface cisterns and plasma membrane of hippocampal neurons
Source: Mol Brain. 2018 Apr 16;11:23. doi: 10.1186/s13041-018-0366-7 (PMC5902880; doi:10.1186/s13041-018-0366-7)
Supplement: Supplementary file 1 — Number of subsurface cisterns (SSC) normalized to per 10 neuronal somas in dissociated hippocampal cultures. (PDF 264 kb) [file 13041_2018_366_MOESM1_ESM.pdf]

Additional file 1. Number of subsurface cisterns (SSC) normalized to per 10 neuronal somas in dissociated hippocampal cultures

	Experiment conditions	Total number of SSC (Types A+B+C) (n = # somas)		SSC with a flattened cistern		
				Type B	Type C	Subtotal (B+C)
**Exp 1**	**Control**	78	(20)	2	0.5	2.5
	**2’ high K^+**	52.5	(20)	3.5	0	3.5
**Exp 2**	**Control**	86.5	(20)	3	0	3
	**2’ high K^+**	57.5	(20)	1.5	0	1.5
**Exp 3**	**Control**	90	(10)	1	4	5
	**2’ high K^+**	39	(19)	2.6	2	4.6
	**30’ recovery**	74	(10)	3	0	3
**Exp 4**	**Control**	185	(11)	5.5	3.6	9.1
	**2’ high K^+**	60	(10)	4	0	4
	**30’ recovery**	184	(11)	2.7	3.6	6.3
**Exp 5**	**Control**	71	(12)	1.7	0	1.7
	**3’ NMDA 30µM**	48.5	(12)	2.5	0	2.5
**Exp 6**	**Control**	67	(10)	5	0	5
	**2’ NMDA 50 µM**	39.5	(17)	2.4	0	2.4
	**30’ recovery**	71	(10)	1	0	1
