# Supplementary material for: Activity-dependent decrease in contact areas between subsurface cisterns and plasma membrane of hippocampal neurons
Source: Mol Brain. 2018 Apr 16;11:23. doi: 10.1186/s13041-018-0366-7 (PMC5902880; doi:10.1186/s13041-018-0366-7)
Supplement: Supplementary file 2 — Average length of subsurface cistern (mean ± SEM in nm) in neuronal somas of dissociated hippocampal neuronal cultures. (PDF 46 kb) [file 13041_2018_366_MOESM2_ESM.pdf]

Additional file 2. Average length of subsurface cistern (mean ± SEM in nm) in neuronal somas of dissociated hippocampal neuronal cultures

	**Control**	**High K^+**	**Recovery**
**Exp 1**	336 ± 31 (100)<br>SD=314	236 ± 12 (108)<br>SD=128<br>P<0.005<br>(Student t test)	-
**Exp 2**	426 ± 40 (84)<br>SD=368	167 ± 11 (75)<br>SD=92<br>P<0.0001<br>(Student t test)	-
**Exp 3**	375 ± 23 (90)<br>SD=217	232 ± 17 (73)<br>SD=148<br>P<0.0001 vs. cont<br>(ANOVA)	383 ± 26(74)<br>SD=221<br>P<0.0001 vs. high K^+
**Exp 4**	281 ± 17 (202)<br>SD=249	139 ± 12 (61)<br>SD=91<br>P<0.0001 vs. cont<br>(ANOVA)	293 ± 17 (204)<br>SD=240<br>P<0.0001 vs. high K^+
**Ranges**	40-2467	27-680	40-1533

	**Control**	**NMDA**	**Recovery**
**Exp 5**	485 ± 54 (88)<br>SD=509	324 ± 24 (58)<br>SD=184<br>P<0.01<br>(Student t test)	-
**Exp 6**	425 ± 37 (67)<br>SD=300	256 ± 24 (67)<br>SD=198<br>P<0.001 vs. cont<br>(ANOVA)	378 ± 35 (71)<br>SD=293<br>P<0.05 vs. NMDA2
**Ranges**	67-3066	40-1066	93-2000

Experiment numbers are the same as in Additional file 1.

(n=number of SSC measured)

SEM (standard error of the mean); SD (standard deviation)
