# Supplementary material for: Activity-dependent decrease in contact areas between subsurface cisterns and plasma membrane of hippocampal neurons
Source: Mol Brain. 2018 Apr 16;11:23. doi: 10.1186/s13041-018-0366-7 (PMC5902880; doi:10.1186/s13041-018-0366-7)
Supplement: Supplementary file 3 — Number of subsurface cistern (SSC) normalized to per 10 pyramidal neuronal somas in the CA1 region of the hippocampus in organotypic slice cultures. (PDF 264 kb) [file 13041_2018_366_MOESM3_ESM.pdf]

**Additional file 3. Number of subsurface cistern (SSC) normalized to per 10 pyramidal neuronal somas in the CA1 region of the hippocampus in organotypic slice cultures**

| | Experiment conditions | Total number of SSC (types A+B+C) (n = # soma) | SSC with a flattened cistern | | |
|---|---|---|---|---|---|
| | | | Type B | Type C | Subtotal (B + C) |
| **Exp 1** | **control** | 81.9 (21) | 10 | 6.2 | 16.2 |
| | **3’ $K^+$** | 29.5 (21) | 1.9 | 4.3 | 6.2 |
| **Exp 2** | **control** | 73.5 (23) | 11.7 | 2.6 | 14.3 |
| | **30” $K^+$** | 44.2 (21) | 6.2 | 6.2 | 12.4 |
| | **2’ $K^+$** | 17.7 (22) | 3.6 | 3.2 | 6.8 |
| | **5’ $K^+$** | 10.6 (17) | 2.9 | 1.2 | 4.1 |
| **Exp 3** | **control** | 122.2 (14) | 1.4 | 3.6 | 5 |
| | **3’ $K^+$** | 23.9 (21) | 2.4 | 1 | 3.4 |
| | **3’ $K^+$+1’ recovery** | 40.9 (21) | 2.9 | 1.9 | 4.8 |
| | **3’ $K^+$+5’ recovery** | 98 (15) | 4 | 3.3 | 7.3 |
| **Exp 4** | **control** | 71.4 (22) | 7.3 | 5 | 12.3 |
| | **3’ $K^+$** | 23.2 (22) | 6.4 | 1.8 | 8.2 |
| | **3’ $K^+$+10’ recovery** | 94.8 (27) | 6.3 | 5.9 | 12.2 |
| | **3’ $K^+$+1hr recovery** | 82.5 (24) | 6.3 | 6.7 | 13 |
| **Exp 5** | **control** | 96.3 (19) | 5.8 | 3.7 | 9.5 |
| | **1’ $K^+$** | 35.7 (21) | 5.7 | 2.4 | 8.1 |
| | **1’ $K^+$+2’recovery** | 50 (13) | 10.7 | 3.8 | 14.5 |
| **Exp 6** | **control** | 94.3 (21) | 6.7 | 4.8 | 11.5 |
| | **1’ $K^+$** | 31.7 (23) | 1.7 | 3.9 | 5.6 |
| | **1’ $K^+$+1’recovery** | 51 (20) | 6.5 | 3.5 | 10 |
| | **1’ $K^+$+5’recovery** | 110.6 (17) | 9.4 | 8.8 | 18.2 |
| **Exp 7** | **control** | 122 (10) | 7 | 6 | 13 |
| | **30” NMDA** | 78.2 (11) | 2.7 | 4.5 | 7.2 |
| | **1’ NMDA** | 59 (10) | 8 | 2 | 10 |
| | **3’ NMDA** | 32.7 (11) | 3.6 | 4.5 | 8.1 |
| | **5’ NMDA** | 16 (10) | 7 | 0 | 7 |
| **Exp 8** | **control** | 136.7 (9) | 4.4 | 6.7 | 11.1 |
| | **30” NMDA** | 99.2 (12) | 7.5 | 4.2 | 11.7 |
| | **1’ NMDA** | 64.5 (11) | 9.1 | 1.8 | 10.9 |
| | **2’ NMDA** | 46 (10) | 5 | 4 | 9 |
