# Supplementary material for: Activity-dependent decrease in contact areas between subsurface cisterns and plasma membrane of hippocampal neurons
Source: Mol Brain. 2018 Apr 16;11:23. doi: 10.1186/s13041-018-0366-7 (PMC5902880; doi:10.1186/s13041-018-0366-7)
Supplement: Supplementary file 4 — Average length of subsurface cistern (mean ± SEM in nm) in pyramidal neuronal somas in the CA1 region of organotypic hippocampal slice cultures. (PDF 45 kb) [file 13041_2018_366_MOESM4_ESM.pdf]

Additional file 4. Average length of subsurface cistern (mean ± SEM in nm) in pyramidal neuronal somas in the CA1 region of organotypic hippocampal slice cultures

	Control	High K^+		Recovery	
**Exp #1**	252 ± 17 (134)<br>SD=192	**3' K^+**	168 ± 15 (65)<br>SD=120<br>P<0.0005<br>(Student t test)	–	
**Exp #2**	272 ± 19 (77)<br>SD=194	**5' K^+**	188 ± 41 (22)<br>SD=190<br>P<0.1<br>(Student t test)	–	
**Exp #4**	258 ± 24 (109)<br>SD=213	**3' K^+**	187 ± 25 (51)<br>SD=179<br>P<0.1, vs. cont<br>(ANOVA)	**3' K^+ +<br>30' recovery**	238 ± 15 (125)<br>SD=167<br>N. S. vs. K^+
**Exp #6**	268 ± 18 (76)<br>SD=155	**1' K^+**	192 ± 16 (71)<br>SD=134<br>P<0.05, vs. cont<br>(ANOVA)	**1' K^+ +<br>5' recovery**	266 ± 19 (118)<br>SD=211<br>P<0.05, vs. K^+
**Ranges**	40-1333	27-933		53-1240	

Experiment numbers are the same as in Additional file 3.

(n=number of SSC measured)

SEM (standard error of the mean); SD (standard deviation)
