# Supplementary material for: Activity-dependent decrease in contact areas between subsurface cisterns and plasma membrane of hippocampal neurons
Source: Mol Brain. 2018 Apr 16;11:23. doi: 10.1186/s13041-018-0366-7 (PMC5902880; doi:10.1186/s13041-018-0366-7)
Supplement: Supplementary file 5 — Percentages of each of the three types of SSC (A, B and C) in neuronal somas before and after depolarization. (PDF 264 kb) [file 13041_2018_366_MOESM5_ESM.pdf]

Additional file 5. Percentages of each of the three types of SSC (A, B and C) in neuronal somas before and after depolarization

		**Type A**<br>**Single stack with an open cistern**	**Type B**<br>**Single stack with a flat cistern**	**Type C**<br>**Double stacks with a flat cistern**
**Dissociated cells**	**Control**	95.5 ± 0.8 %	3.4 ± 0.9 %	1.2 ± 0.7 %
	**High K^+**	93.1 ± 1.9 %	5.7 ± 1.0 %	1.3 ± 1.3 %
**Slice cultures**	**Control**	84.8 ± 3.7 %	9.9 ± 3.1 %	5.3 ± 1.2 %
	**High K^+**	72.7 ± 5.8 %	16.1 ± 4.8 %	11.2 ± 3.2 %

For dissociated cells, *% values calculated from data in Additional file 1 (exp 1-4). No statistical significant differences between control and high K^+ in all categories (paired t test).*

For slice cultures, % values calculated from data in Additional file 3 (exp 1-4). Upon depolarization with high K^+, there was a near significant decrease (P~ 0.06, paired t test) in percentages in SSC with an open cistern (type A), and a near significant increase (P~ 0.06, paired t test) in percentages in SSC with a flattened cistern (Types B+C).
