# Supplementary material for: Activity-dependent decrease in contact areas between subsurface cisterns and plasma membrane of hippocampal neurons
Source: Mol Brain. 2018 Apr 16;11:23. doi: 10.1186/s13041-018-0366-7 (PMC5902880; doi:10.1186/s13041-018-0366-7)
Supplement: Supplementary file 6 — (A) Total number of SSC and (B) Subtotal number of SSC with a flattened stack of ER in pyramidal neuronal somas in the CA1 region of hippocampal slice cultures. Number of SSC in control samples normalized to per 10 neuronal somas. Values for other conditions normalized to % of controls. (PDF 52 kb) [file 13041_2018_366_MOESM6_ESM.pdf]

Additional file 6. (A) Total number of SSC and (B) Subtotal number of SSC with a flattened stack of ER in pyramidal neuronal somas in the CA1 region of hippocampal slice cultures. Number of SSC in control samples normalized to per 10 neuronal somas. Values for other conditions normalized to % of controls.

exp		cont	30" K^+	1' K^+	2' K^+	3' K^+	5' K^+	K^++ 1'rec	K^++ 2'rec	K^++ 5'rec	K^++ 10'rec	K^+ + 1h rec
1	A. Total SSC	81.9				36%						
	B. sub flat	16.2				38%						
2	A. Total SSC	74.8	60%		24%		13%					
	B. sub flat	14.3	87%		48%		27%					
3	A. Total SSC	122.2				20%		33%		80%		
	B. sub flat	6				57%		80%		122%		
4	A. Total SSC	70.9				26%					134%	116%
	B. sub flat	12.3				67%					100%	106%
5	A. Total SSC	96.3		37%					50%			
	B. sub flat	9.5		85%					153%			
6	A. Total SSC	84.3		38%				60%		131%		
	B. sub flat	11.5		49%				87%		158%		

Experiment numbers are the same as in Additional file 3.

rec (recovery in control medium)
